# Left-Sided Gall Bladder: An Anatomic Rarity and Its Implications

**DOI:** 10.7759/cureus.39754

**Published:** 2023-05-30

**Authors:** Moses Amarjothi Joacquim, Kannan Devvygounder, Anbalagan P

**Affiliations:** 1 Surgery, United Lincoln NHS Trust, Lincoln, GBR; 2 Surgical Gastroenterology, Saveetha Medical College, Chennai, IND

**Keywords:** laparascopic surgery, biliary anatomy, modifications in technique, laparoscopic cholecystectomy, left sided gallbladder

## Abstract

Left-sided gall bladder (LGB) is a rare anomaly seldom encountered by surgeons in clinical practice. Accurate preoperative diagnosis is rare due to the atypical localisation of pain in the right hypochondrial quadrant and the rarity of occurrence. This feature poses intraoperative challenges that mandate quick improvisation. Hence, all surgeons should gain knowledge of left-sided gall bladders, which can be associated with the risk of biliovascular injuries compared to normally positioned gall bladders. We present an interesting case of the intraoperatively diagnosed left-sided gall bladder, where a few minor modifications in laparoscopic technique could salvage the situation with marked improvement in surgical ease and consequent outcomes.

## Introduction

Left-sided gall bladder (LSG) is a rare anatomic anomaly that can perplex surgeons and create challenges in surgical management. A robust knowledge of biliary surgical anatomy, types of left-sided gall bladders, associated anomalies, and modifications of the surgical technique can help tide over intraoperative difficulties and achieve good surgical outcomes.

## Case presentation

A 35-year-old woman reported to our hospital with a three-day history of acute epigastric pain in the right hypochondria associated with vomiting. The clinical examination revealed mild right hypochondrial tenderness with no elevation of temperature. Laboratory investigations showed normal white blood cell counts, alkaline phosphatase, and bilirubin.

**Figure 1:**
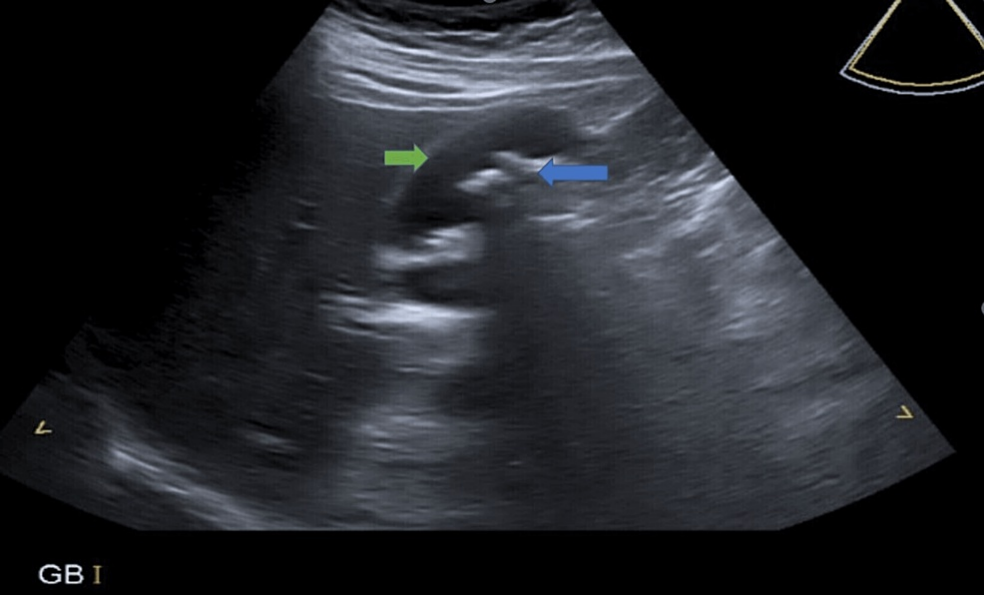
Ultrasound showed a thickened gall bladder wall (green arrow) with multiple stones in gall bladder (blue arrow)

Abdominal ultrasound showed a gall bladder with a thickened wall of size 5 mm, multiple stones in the gall bladder of size 5 mm each, normal common bile duct (CBD) (6 mm), and spleen in the left hypochondrium (Figure 1). No other pathology was identified. Chest radiography revealed no dextrocardia.

**Figure 2:**
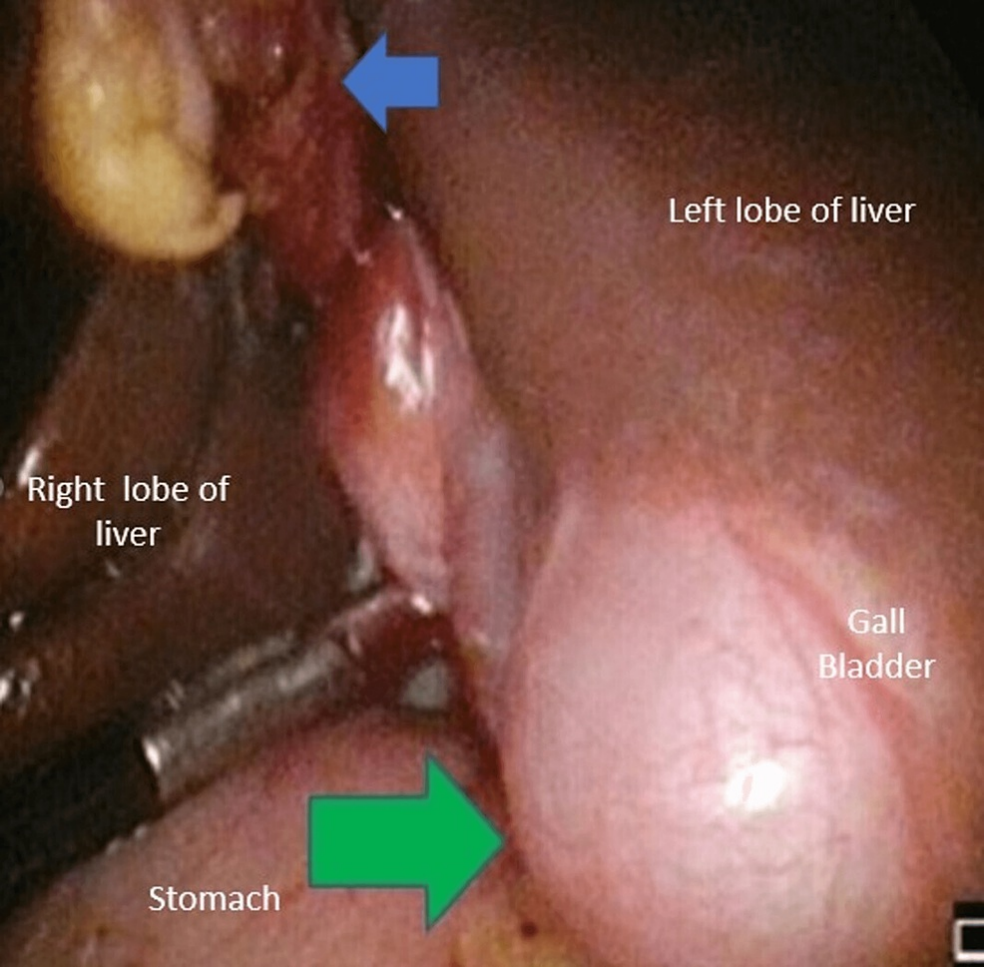
Sinistroposition left-sided gall bladder The gall bladder is seen along segment 3 of the liver (green arrow) to the left of the falciform ligament (FL) (blue arrow).

**Figure 3:**
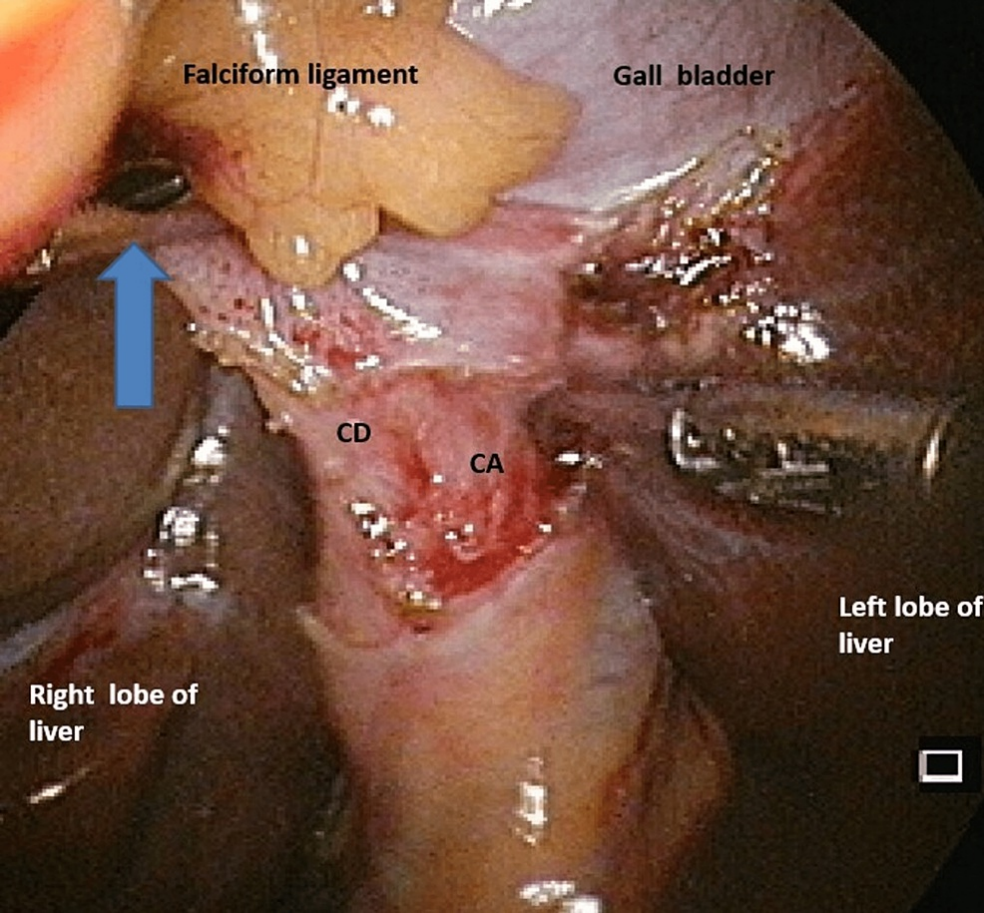
Critical view of safety achieved delineating the cystic duct (CD) and cystic artery (CA) entering the gall bladder The epigastric trocar is seen through a window in the falciform ligament (FL) facilitating the view of the left-sided gall bladder.

The patient was prepared for laparoscopic cholecystectomy. At operation and after umbilical port (10 mm) insertion, a left-sided gall bladder (LSG) was visualised to the left of the falciform ligament (Figure 2). Consequently, a few minor modifications were made to dissection using the fundus-first approach to achieve the critical view of safety (Figure 3). A falciform window with accessory port incision and left-sided elevation was done to improve surgeon ergonomics and increase the ease of surgery in the Calot’s triangle. The patient was discharged the next day with an uneventful recovery.

## Discussion

The gall bladder is characteristically located in the gall bladder fossa, which is between Couinaud liver segments 4b and 5. Sometimes, there may be aberrations, including ectopic positions: intrahepatic, transverse, retro-displaced, and left-sided [1]. Left-sided gall bladders are defined as those to the left of the falciform ligament (FL), between Couinaud segments 3 and 4b [2]. The first description of the left-sided gall bladder was by Cirla and De Vecchi in 1965 [3]. The first successful laparoscopic cholecystectomy in a left-sided gall bladder with situs inversus totalis was performed in 1991 [4]. 

**Figure 4:**
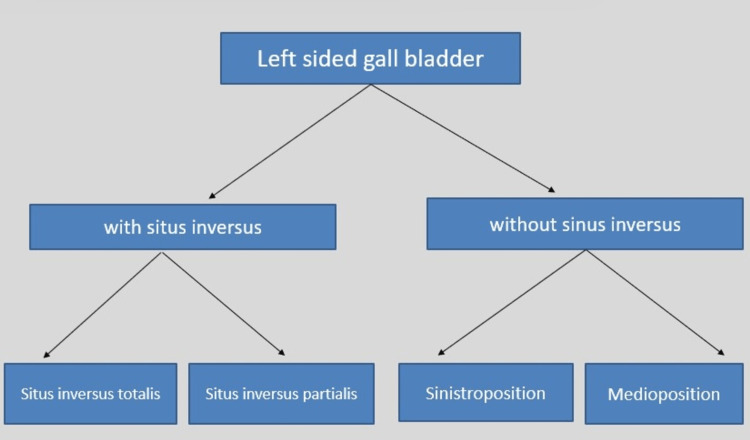
Classification of left-sided gall bladders

Left-sided gall bladders are broadly classified based on the association with situs inversus (Figure 4). Those without situs inversus are of two types based on the location of the gall bladder fossa to the falciform ligament. They include sinistroposition and medioposition.

Sinistroposition (true left-sided gall bladder)

It is located to the left of the falciform ligament between segments 4b and 3 (like our case). The aetiology could be either migration of the nascent gall bladder to the left of the falciform ligament or duplication of the gall bladder on the left with regression of the one on the right in the gall bladder fossa [5].

Medioposition (gall bladder with right-sided falciform ligament or false left-sided gall bladder)

It is located to the right of the falciform ligament. The first case was reported in 1886, and the literature shows an incidence of 0.1-1.2% [6]. The aetiology could be atrophy of the normally left-dominant falciform ligament, creating an illusion of a left-sided gall bladder due to the right-dominant falciform ligament [7].

Anomalies

Both sinistroposition and medioposition gall bladders may show anomalies, which may be associated with biliovascular injuries [8]. Sinistroposition gall bladders mainly exhibit biliary anomalies, with the most common finding being atrophy of segment 4 of the liver. Other anomalies include abnormal origin, course of the cystic artery, and insertion of the cystic duct on the right side of the common hepatic duct (CHD), which manifests as structures crossing the entire hilar area. Another notable anomaly is the embedding of the gall bladder in the ectopic gall bladder bed, which may necessitate the usage of energy devices to control bleeding.

The medioposition gall bladder is also associated with portal vein anomalies, most notably trifurcation of the portal vein with biliary anomalies and segment 4 atrophy [9]. This feature is crucial in planning surgeries, such as hepatectomy and cholecystectomy, done commonly in most surgical centres. Therefore, both sinistroposition and medioposition gall bladders can be considered markers for portal and biliary vascular anomalies, and a thorough evaluation, if preoperative diagnosis is feasible.

Clinical features/imaging

Unless there is a high index of clinical suspicion, left-sided gall bladders are likely to be missed preoperatively. Routine preoperative imaging with ultrasound and CT is dismally inaccurate, with sensitivity as low as 16.3%. As up to 75.05% of patients may have right upper quadrant pain, as is the norm in normally positioned gall bladders, an index of diagnostic suspicion for ectopic gall bladder may not be commonplace. Reports suggest that the transposition of the gall bladder is not associated with the transposition of the nerve fibres away from the right hypochondrium leading to this discrepancy. magnetic resonance cholangiopancreatography (MRCP) and angiography are recommended to increase the preoperative accuracy of diagnosis [10].

Treatment

The treatment of choice in cholecystitis involving left-sided gall bladder is still laparoscopic cholecystectomy, with a few modifications. Following the basic tenets of laparoscopic surgery such as safe and meticulous dissection, ligation of structures close to the gall bladder, low threshold for conversion to open cholecystectomy, and minimal use of diathermy, is a must. Energy devices like harmonic scalpels may be necessary for impacted gall bladder in the gall bladder fossa [11]. Intraoperative cholangiography may be required to delineate the biliary anatomy and minor changes in port placement.

These port changes include using 2 mm ports, placement of accessory ports on the left, alteration in the position of the conventional epigastric port to the left subcostal region, use of Palmer’s point for port placement and ‘mirroring’ ports like on the right [12]. Changes in patient and surgeon position include the French position (surgeon position between the legs of the patient ), left side elevation of the patient, and falciform ligament modifications, such as window creation (as in our case) or falciform lift to aid easy dissection and provide an unimpeded view of the after epigastric port placement [13]. Two-stage procedure (cholecystostomy followed by cholecystectomy) is preferable for surgeries in areas of resource scarcity, severe inflammation of the Calots, or diagnostic difficulty [14].

## Conclusions

Left-sided gall bladders are a rare anomaly requiring intraoperative awareness for satisfactory management. Hence, knowledge of treatment options involving both types of left-sided gall bladders is crucial in preventing biliovascular injuries and obtaining good patient outcomes. Sinistroposition and medioposition of left-sided gall bladders can be associated with portal and bilovascular anomalies, necessitating comprehensive evaluation before contemplating surgery.
